# Isolation, identification and antimicrobial susceptibility of *Pantoea* (*Enterobacter*) *agglomerans* isolated from consumed powdered infant formula milk (PIF) in NICU ward: First report from Iran

**Published:** 2013-09

**Authors:** Jalal Mardaneh, Mohammad Mehdi Soltan Dallal

**Affiliations:** 1 Department. of Pathobiology, School of Public Health, Tehran University of Medical Sciences, Tehran, Iran; 2 Prof. Alborzi Clinical Microbiology Research Center, Shiraz University of Medical Sciences, Shiraz, Iran; 3 Food Microbiology Research Center, Tehran University of Medical Sciences, Tehran, Iran

**Keywords:** Powdered infant formula milk (PIF), neonatal intensive care unit (NICU), *Pantoea agglomerans*, antimicrobial susceptibility

## Abstract

**Background and Objectives:**

*Pantoea agglomerans* is a Gram-negative rod in the *Enterobacteriaceae* family. It is reported as both commensal and opportunistic pathogen of animals and humans. This organism is potential candidates as powdered infant milk formula-borne opportunistic pathogen. The aim of our study was to perform isolation, identification and antimicrobial susceptibility pattern of *Pantoea* (*Enterobacter*) *agglomerans* strains isolated from consumed powdered infant formula milk (PIF) in NICU ward.

**Materials and Methods:**

A of total 125 powdered infant formula milk (PIF) samples were purchased from hospital drug stores between June 2011 to March 2012. *P. agglomerans* was isolated according to FDA method. For final confirmation, biochemical tests embedded in the API-20E system were used. The drug susceptibility test was performed using the disc diffusion method according to CLSI guidelines.

**Results:**

Out of the 125 samples investigated, 8 (6.4%) samples were positive for *P. agglomerans* and these were uniformly susceptible to tigecycline, chloramphenicol, cefepime, levofloxacin, minocycline and colistin. Fifty percent of isolates were resistant to cefotaxime, moxifloxacin, cotrimoxazole and ticarcillin.

**Conclusion:**

Controlling the primary populations of *P. agglomerans* during the PIF production process and preventing post processing contamination, by using suitable microbiological guidelines, is accessible. Sanitary practices for the preparation of infant formula in both the home and hospitals should be carefully controlled.

## INTRODUCTION

*Pantoea agglomerans* (formerly *Enterobacter agglomerans*) is a motile peritrichous, non-sporforming, Gram-negative aerobic bacilli in the *Enterobacteriaceae* family (1, 2). It is commonly found in the ecological niches such as water, soil, sewage, seeds, vegetables, feculent material and foodstuffs, as well as reported as both commercial and opportunistic pathogens of animals and humans (3, 4). This opportunistic pathogen isolated from clinical specimens including blood, wounds, urine, throat, and internal organs (5).

*P. agglomerans* is recognized as a plant pathogen. In the mid-1960s it was identified in nosocomial infections (6, 7). *P. agglomerans* is the most frequent species associated with human infections (1, 8). Hospital outbreaks due to contamination of anesthetic agent propofol, blood products, parenteral nutrition, and transference tubes used for intravenous hydration have been demonstrated (8, 9, 10).

*P. agglomerans* has been implicated in pneumonia, wound infections, septicemia, bacteremia, urinary tract infection, meningitis, lung and brain abscess, septicemia, osteomyelitis, septic arthritis, peritonitis and colelithiasis. The organism is generally regarded as opportunistic, of low virulence, low degree of toxicity and with little intrinsic invasiveness but can cause infection even in the healthy individuals with immunocompetent system (11, 12).

*P. agglomerans* is causative agent of infection in children and elderly persons. It can cause bacteriemia, often in association with more-conventional pathogens, in children with underlying conditions including indwelling catheters (1). *P. agglomerans* is also responsible for outbreak in a neonatal intensive care unit (10). It has previously been described as a cause of bacteremia or sepsis in neonates less than 30 days of age (13, 14).

In recent years a remarkable increase in nosocomial infections has been reported especially in neonatal intensive care unit (NICU), intensive care units (ICU) and oncology departments. Underlying diseases, low-birth-weight, immunocompromised immune system, cancer chemotherapy, and intravenous catheterization can be predisposing factors in cases of infections due to unusual microorganisms, including *P. agglomerans* in neonates. Clinical manifestations are often misleading and, in some circumstances, it may be difficult or even impossible to distinguish the source of the infection. Enterobacteriaceae family members such as *P. agglomerans* are potential candidates as powdered infant milk formula-borne pathogens (15, 16).

Neonates and young children are exclusively vulnerable to infections caused by foodborne pathogens. Contamination of powdered infant formula with *P. agglomerans* will be associated with the development of disease among neonates. Therefore, the microbiological safety of powdered infant formula milk (PIF) is of most importance. Because PIF is not a sterile product, it is an excellent medium to support bacterial growth. Bovine milk and plant materials are essential ingredients of PIF and a potential source of various bacteria that are pathogenic to neonates and adults. The aim of this study was to isolate *Pantoea* (*Enterobacter*) *agglomerans* from consumed PIF in NICU ward in Tehran hospitals and to determine the antimicrobial susceptibility pattern of these isolates.

## MATERIALS AND METHODS

### Sampling

A cross-sectional study was carried out on 125 powdered infant formula milk (PIF) samples purchased from hospital drug stores between June 2011 to March 2012.

### Isolation and Identification

For isolation of *P. agglomerans* from samples, the PIF cans were surface sterilized with 70% ethanol (Merck Co.) and were opened in a laminar flow cabinet. Samples were taken from each product under aseptically conditions. *P. agglomerans* was isolated according to the FDA method (17, 18). We prepared 3 Erlenmeyer flask each of sterile distilled water (pre-warmed to 45°C) at 9, 90 and 900 ml containing 1, 10 and 100 g of PIF, respectively. After the PIF was completely mixed and dissolved in distilled water, it was incubated at 37°C for 18-24 h. Following the incubation, 10 ml of each sample was added to 90 ml of Enterobacteriaceae enrichment (EE) broth medium and placed at 37°C for 18-24 h. After incubation, a loopful of the enrichment culture was streaked onto duplicate violet red bile glucose agar (VRBGA) plates and cultured at 37°C for 18-24 h. A total of 4 suspicious colonies were picked from each VRBGA plate and pure culture was performed. For detection of non-lactose fermenting isolates, presumptive colonies were streaked onto MacConkey agar and incubated at 37°C for 72 h. The API-20E biochemical kit system (Bio-Mérieux) and manual biochemical tests were used to identify the organism accoding to the manufacturer instruction (5). For long term storage, the purified isolates were saved in tryptic soy broth (TSB) with 20% glycerol (Merck Co.) at -20°C.

### Antibiotic susceptibility testing

Antibiotic susceptibility testing was done using Kirby-Bauer disk diffusion method on Mueller Hinton agar according to CLSI guidelines (19). Antimicrobial agents used in this study, were ampicillin (10 µg), piperacillin (100 µg), piperacillin-tazobactam (100/10 µg), mezlocillin (75 µg), carbenicillin (100 µg), ticarcillin (75 µg), ceftazidime (30µg), cefotaxime (30 µg), ceftriaxone (30 µg), cefepime (30 µg), imipenem (10 µg), meropenem (10µg), aztreonam (30 µg), streptomycin (10 µg), amikacin (30 µg), gentamicin (10 µg), tobramycin (10 µg) tetracycline (30 µg), minocycline (30 µg), tigecycline (15 µg), chloramphenicol (30 µg), ciprofloxacin (5 µg), levofloxacin (5 µg), moxifloxacin (5 µg), colistin (25 µg), trimethoprim-sulfamethoxazole (1.25/23.75 µg) and nalidixic acid (30 µg).

## RESULTS

Out of the 125 PIF samples investigated, 8 (6.4%) samples were positive for *P. agglomerans*. The Gram staining showed Gram negative rods. On VRBGA agar purple/pink colored colonies, and on MacConkey agar convex, smooth, punctuate, umbilicated lactose fermenting glistening colonies were grown. Isolates were catalase positive, oxidase negative, motile and produced other biochemical reactions, which are characteristic of *P. agglomerans*. All eight isolates from powdered infant milk formula samples were uniformly susceptible to tigecycline, chloramphenicol, cefepime, levofloxacin, minocycline and colistin. Fifty percent of the isolates were resistant to cefotaxime, moxifloxacin, cotrimoxazole and ticarcillin, 62.5% to carbenicillin, and 37.5% to ampicillin, piperacillin and mezlocillin (Table 1).

**Table 1:** Antimicrobial susceptibility patterns of *P. agglomerans* strains isolated from PIF. (N = 8)

Antibiotic	Sensitive (%)	Intermediate (%)	Resistant (%)
Tigecycline	8 (100)	-	-
Amikacin	5 (62.5)	2 (25)	1 (12.5)
Ampicillin	1 (12.5)	4 (50)	3 (37.5)
Aztreonam	6 (75)	1 (12.5)	1 (12.5)
Cefotaxime	3 (37.5)	1 (12.5)	4 (50)
Gentamicin	6 (75)	2 (25)	-
Meropenem	5 (62.5)	2 (25)	1 (12.5)
Mezlocillin	3 (37.5)	2 (25)	3 (37.5)
Moxifloxacin	4 (50)	-	4 (50)
Nalidixic acid	5 (62.5)	2 (25)	1 (12.5)
Streptomycin	6 (75)	2 (25)	-
Tetracycline	7 (87.5)	1 (12.5)	-
Ticarcillin	4 (50)	-	4 (50)
Chloramphenicol	8 (100)	-	-
Ceftazidime	6 (75)	1 (12.5)	1 (12.5)
Ciprofloxacin (CIP)	7 (87.5)	1 (12.5)	-
Cefepime (CPM)	8 (100)	-	-
Imipenem (IMI)	6 (75)	2 (25)	-
Levofloxacin (LEV)	8 (100)	-	-
Minocycline (MN)	8 (100)	-	-
Piperacillin (PRL)	5 (62.5)	-	3 (37.5)
Piperacillin-tazobactam (PTZ)	5 (62.5)	1 (12.5)	2 (25)
Carbenicillin (PY)	1 (12.5)	2 (25)	5 (62.5)
Tobramycin (TN)	7 (87.5)	1 (12.5)	-
Cotrimoxazole (TS)	4 (50)	-	4 (50)
Amoxicillin (A)	2 (25)	4 (50)	2 (25)
Colistin (CO)	8 (100)	-	-

## DISCUSSION

*P. agglomerans*, until recently known as *Enterobacter agglomerans* and it's new nomenclature is not yet widely in use (20). It is an opportunistic pathogen and, when introduced into the organs of humans or other animals, may cause severe and occasionally fatal infections. The most serious infections are in individuals with underlying diseases and in the young persons (21). Since clinical reports involving *P. agglomerans* are typically of polymicrobial nature, confirmed virulence of *P. agglomerans* is difficult to reveal. Infections caused by this organism often involve patients that are already affected by diseases of other origin, and isolates are rarely conserved for confirmatory analysis (22). *P. agglomerans* is ubiquitous in nature and it has been isolated from a wide variety of ecological niches and from different kinds of specimens from humans and animals (21). *P. agglomerans* is mostly isolated from powdered infant formula in developed and developing countries through the world (15, 16, 23).

To our knowledge, there is no previous report on the isolation and identification of *P. agglomerans* from powdered infant milk formula milk in Iran. Samples belonged to different companies. In this study, we demonstrated that this product is contaminated with *P. agglomerans*. From our results we conclude that contamination of infant milk formula with multi drug resistance bacteria such as *P. agglomerans* is important. The inherent capability of this organism to remain viable and grow well at room temperature may contribute to such contamination. It is important to mention that some bacteria under stress conditions (e.g. pasteurization process) may enter into the viable, but nonculturable state.

Powdered milk infant formula is not a sterile product and may be intrinsically or extrinsically contaminated with various bacteria that can cause serious disease in infants. Even healthy babies may sometimes acquire such foodborne infections. Neonates and high risk groups of individuals (e.g. elderly persons, immunosuppressed patients) are particularly assailable to foodborne pathogens, including *P. agglomerans* (24–26). Care givers in hospital neonatal units should be constantly alert to the fact that powdered infant formula products are not sterile and may be colonized by bacterial organisms. Therefore, the use of hygienic measures during preparation and reconstitution are essential. To decrease the risk of foodborne illness in neonates feed infant formula, recommendations have been made for the preparation, storage and handling of PIF. In addition, infant formula producers must accomplish guidelines aimed at decreasing the risks of product contamination with foodborne pathogens. Controlling the primary populations of *P. agglomerans* during the PIF production process and preventing post processing contamination, by using suitable microbiological guidelines, is accessible. Sanitary practices for the preparation of infant formula in both the home and hospitals should be carefully controlled.

Powdered infant milk formula is not always sold in the country where it is manufactured and also possible that some or all formula ingredients are imported. However it may be supposed that the contamination by members of the Enterobacteriaceae family occur after the pasteurization of the infant formula products. Howsoever the mode of transmission is not always clear, but two main routes by which *P. agglomerans* can enter PIF are including (1) internal (intrinsic) contamination (e.g. through contaminated ingredients added after drying or from the processing environment following drying steps and before packaging), and (2) external (extrinsic) contamination of the PIF during reconstitution and handling.

Powdered infant milk formula without members of the Enterobacteriaceae might propose additional protection to the neonates and especially to the low birth weight premature babies if some multiplication during the preparation, storage or handling in contaminated infant formula does occur. There are no active surveillance systems for *P. agglomerans* in developed and developing countries. Given the limited scope and recent performance of active surveillance systems, many years of continuous surveillance will be required to establish a dependable estimate of *P. agglomerans* prevalence in PIF and other products.

There is very little known about virulence factors and pathogenicity of *P. agglomerans*. Complementary studies are necessary to clarify the possible role of *P. agglomerans* as a food contaminant, in common NICU infections and high risk groups including immunocompromised individuals.
